# Medical Students' Attitudes Toward Non-Adherent Patients Before and After a Simulated Patient-Role Activity and Small-Group Discussion: Revisited

**DOI:** 10.7759/cureus.576

**Published:** 2016-04-19

**Authors:** Angela DelPrete, Christin Giordano, Analia Castiglioni, Caridad Hernandez

**Affiliations:** 1 College of Medicine, University of Central Florida College of Medicine; 2 Internal Medicine, University of Central Florida College of Medicine; 3 Practice of Medicine, University of Central Florida College of Medicine

**Keywords:** empathy, medical student, jefferson scale of physician empathy

## Abstract

Introduction

This study seeks to explore whether the documented decline in medical student empathy can be prevented or slowed using simulated patient-role activities and small-group discussions about the patient experience of living with a chronic illness.

Methods

First-year students (M1, *n *= 118) at the University of Central Florida College of Medicine (UCFCOM) participated in a simulated patient-role activity resembling the experience of a patient with Type 2 diabetes mellitus. The activity included taking daily “medication,” participating in moderate exercise, and maintaining a low carbohydrate diet. At the end of the simulated patient-role activity, students took part in a small-group discussion about their experiences. Students completed the Jefferson Scale of Physician Empathy: Student Version (JSPE:S) before and after the activity. Additionally, fourth-year students (M4) at UCFCOM completed the JSPE:S to serve as the control, as this class completed the curriculum without any simulated patient-role activities.

Results

A total of 86 responses out of 118 possible M1 participants (73% response rate) were received. Of these, 62 surveys were completed and were therefore used for statistical analysis. A dependent sample t-test revealed no statistically significant increase on pre-activity (*M *= 111.15, *SD *= 8.56) and post-activity (*M *= 111.38, *SD *= 9.12) empathy scores (*p *= .78). A positive correlation was revealed to exist between pre- and post-activity empathy scores (*r *= 0.72, *p *< 0.001). Empathy comparisons for the full sample M1 post-activity results (*n *= 62) and the M4 results (*n *= 16, *M *= 106.56, *SD *= 10.61) revealed no statistically significant difference (*p* = .11).

Discussion

Although previous authors have shown that patient role-playing activities, such as those performed in this study, should maintain and/or increase empathy in medical students, our findings suggest that on a short-term scale, empathy levels were not affected by the activity.

## Introduction

### Empathy in the medical setting

An empathic physician is one who can maintain their objectivity while understanding the subjectivity of a patient’s experience. She or he can remove the patient from the textbook algorithms or stereotypes of their respective disease in order to establish a more personalized connection with the patient. This connection, in turn, provides the foundation for more effective patient-physician communication, which may result in improved patient care [1].^ ^Recent literature supports the notion that medicine relies just as heavily on physician empathy as it does on clinical expertise for effective patient care. For example, Jin, et al. and Hojat, et al. demonstrate those patients are more likely to follow treatment recommendations if they feel that these recommendations are coming from a physician who is understanding [2-3]. In turn, assessments of physicians’ perceptions of patients and their adherence to clinical advice revealed that these perceptions can impact overall clinical judgment, including prescribing patterns and how closely a physician follows established guidelines [4-7]. For example, Wong, et al. found that providers, who indicated that patient adherence plays an important role in their decision to prescribe protease inhibitors to HIV patients, prescribed the drugs later in the treatment plan to Latinos, women, and poor patients compared to physicians who did not cite patient adherence as having an impact on prescribing practices [4]. Additionally, Porter, et al. revealed that perceptions of a patient’s physical or mental status influenced how strictly a physician followed acute coronary syndrome guidelines [5]. Understanding how physician empathy can impact clinical decision-making has the potential to positively influence patient care, leading to more beneficial patient outcomes and creating an overall more benevolent healthcare system. Therefore, incorporating educational activities that serve to promote and maintain empathy during the medical school experience is of utmost importance.

### Teaching empathy to future physicians

Several studies have noted a sharp decline in empathy from medical students’ first year (M1) to their fourth year (M4) [8]. Perhaps in response to results such as these, the Association of American Medical College’s Medical School Objectives Project now includes empathy as one of the necessary objectives of a medical school curriculum, and not surprisingly so, considering the impact that empathy can have on patient outcomes [9].

Unfortunately, medical students are quickly socialized to have negative perceptions towards patients labeled as “non-compliant”. Students in focus groups revealed that patients whose ailments were perceived as “their own fault” (e.g., obesity) were considered to be acceptable targets for “derogatory and cynical humor” [10]. Similar studies have clarified the importance of teaching empathy throughout the entirety of medical education [11]. What is less clear, however, is how to effectively teach empathy.

Despite several definitions of empathy, a common theme is the blurring of differences between ourselves and others in order to more fully understand the experience of another person. Therefore, a seemingly-effective teaching tool presents itself in the form of a simulated patient-role activity. In a breakthrough study, participants either assumed the role of a wheelchair-bound patient or observed the participation of a colleague. Both the direct participants and the observers reported more positive perceptions towards patients with disabilities immediately following the activity and four months later. The authors of this study explain that the improved attitudes are most likely the result of increased empathy levels [12].

Addressing empathy may involve other teaching methods as well. For example, all 12 faculty members participating in a survey about the role of empathy in medical curricula expressed that teaching empathy is necessary and agreed that simulated patient-role activities followed by debriefs were the most effective ways to teach and to develop empathy [13]. A review of nine different types of educational interventions used in attempts to teach empathy as part of medical school curricula concluded that patient-simulation activities and those supplemented by communication skills training (i.e., small-group discussions) showed significant increases in measured empathy [14].

Finally, one must ask whether improved attitudes towards one particular patient group (e.g., diabetics) following an educational intervention translate to changes towards a more inclusive group (i.e., non-adherent patients). Batson, et al. ultimately suggested that they in fact do. In their study, groups of undergraduate students watched fictional videos either of a homeless individual or of a person with AIDS. Students who were asked to consider the interviewee’s emotions reported increased levels of empathy not only towards the person but towards the stigmatized group as a whole. The results of this study suggest that increased empathy levels towards a particular patient group that struggles with therapy compliance may translate to increased empathy levels towards all non-adherent patients [15].

Currently, professionalism, including empathy, is a component of the Practice of Medicine module at the University of Central Florida College of Medicine. This study seeks to incorporate a simulated patient-role element, along with a small-group discussion, in order to evaluate first-year medical students’ empathy levels and attitudes about treatment adherence and determining whether these initial perceptions change following participation in the activity. In the yet unpublished pilot study conducted by Christin Giordano, participants were randomly assigned to one of three different patient scenarios reflecting affliction with either multi-drug resistant tuberculosis, hypertension, or Type 2 diabetes mellitus. Of the three, the patients asked to follow instructions for a patient with diabetes mellitus showed the most significant increase in empathy levels following the role-playing activity and small-group discussion, and these results serve as the foundation for this study. This study aims to lay the groundwork for a longitudinal study to track the long-term effects of the activity throughout the students’ four years of medical school. It also aims to compare these results to data from the control group: rising fourth-year medical students who have completed the standard Practice of Medicine curriculum without the addition of a patient role-playing activity.

We hypothesize those first-year medical students who participate in an activity in which they are required to assume the role of a patient will report more empathetic scores overall on the Jefferson Scale of Physician Empathy: Student Version (JSPE:S) than students in the control group.

We aim to gauge the efficacy of educational patient-role scenarios on maintaining medical student empathy when compared with a curriculum without such activities. We hope to present this data in an effort to improve undergraduate medical education on a national level with the ultimate goal of educating medical students to become both competent physicians and empathetic caretakers.

## Materials and methods

### Participants

Informed consent was obtained from all participants taking part in the study. All first-year medical students enrolled in the UCFCOM Practice of Medicine module were required, per module curriculum, to participate in the simulated patient-role activity (unless the student was physically unable to perform physical activity, to adhere to a low-sodium diet, or to consume the candy "medication") and small-group discussion. Participation in the JSPE:S survey was optional and the decision to participate had no impact on module grades. First-year students were notified of both the required simulated patient-role experience and the opportunity to participate in the survey one week prior to the previously-scheduled required class on March 31, 2014. 

There were 119 first-year students enrolled in the course but the principal investigator was excluded, and thus, a maximum of 118 students participated in the simulated patient-role activity. A total of 86 responses to the JSPE:S were received from the M1 class (73% response rate). Of these, 62 students completed both the pre-activity and post-activity surveys and, thus, 62 surveys were used for statistical analysis. M1 survey participants who completed both pre-activity and post-activity surveys consisted of 26 males and 36 females.

During orientation week in May 2014, M4 students were notified of the opportunity to participate in the JSPE:S survey. A total of 16 responses were received from the M4 class (21% response rate; total = 78 students). Survey participants consisted of seven males and seven females (two participants declined to answer).

An incentive of $5 was offered for completion of both the pre-activity and post-activity surveys for the experimental group (M1) and for completion of the single survey for the control group (M4). The study received approval from the University of Central Florida Institutional Review Board (protocol #SBE-14-09959) on February 20, 2014.

### Procedure

In the week prior to the simulated patient-role activity that began on March 31, 2014, M1 students completed the Jefferson Scale of Physician Empathy: Student Version (JSPE:S) via Qualtrics, a web-based survey platform (Qualtrics, Provo, UT), to establish baseline empathy scores.

Students in the experimental group (M1) participated in a simulated patient-role activity, which was administered as a required component of the Practice of Medicine module by the module director and the principal investigator. Students were asked to follow physician instructions that a patient with diabetes mellitus Type 2 may be required to follow. Instructions for the scenario, as described below, are attached as Appendix C: Diabetes Mellitus Patient Scenario. The instructions include requirements for a combination of “medication” and lifestyle changes (exercise and diet). Students who were unable to participate due to physical limitations, such as illness or disability that would impair their ability to exercise or to adhere to a low-sodium diet, had full discretion to leave the study at any time without consequence. Of note, no students required accommodation or met the exclusion criteria. At the beginning of the activity, students were provided with instructions for the activity and two (2) pill bottles each filled with the required supply of “medication”.

Medications included two (2) pill bottles, one containing a four-day supply of M&Ms and one containing a four-day supply of Tic Tacs. The M&Ms were taken twice a day and the Tic Tacs were taken once a day before breakfast, consistent with dosing instructions for metformin and glyburide, respectively [16-17]. The pill bottles contained instructions for taking the “medications” as well as common side-effects. In addition, students were asked to participate in moderate exercise for twenty (20) minutes two-to-three times over the course of the regimen and to maintain a low carbohydrate diet (less than 65 grams of carbohydrates per meal with three meals a day).

Upon completion of their simulated patient-role activity, students met in their pre-assigned module small groups for a discussion regarding their individual experiences. This small group session was a required portion of the Practice of Medicine module. Group leaders consisted of eight faculty members who were identified by the module director. They were given Appendix D: Faculty Orientation and were instructed to ask the following questions to prompt discussion: *What surprised you about your experience? What was most easy and most difficult about following your regimen and why?  What would have helped you have better adherence?* The discussion group lasted no longer than two hours. Student responses and comments from the discussion groups can be found in the Discussion section and attached as Appendix E: Anonymous Student Comments. The students who had chosen to participate in the survey then completed the post-activity survey (See Appendix B: Post-activity Student Questionnaire) in the manner described for the pre-activity survey.

The control group consisted of fourth-year medical students (M4) who completed the Practice of Medicine course without any activity during their first and second years of medical school. Therefore, there was only one (1) survey for the M4 class to complete (vs. pre-activity and post-activity surveys for the M1 class). All completed surveys were de-identified by the Office of Planning and Knowledge Management prior to release to the principal investigator.

### Instruments

Data collected included the JSPE:S results and gender.

The Jefferson Scale of Physician Empathy: Student Version (JSPE:S; see Appendix A: Pre-Activity Student Questionnaire) is a 20-item Likert scale intended to measure empathy in medical students. This scale was developed in 2001 at Jefferson Medical College and has since been used in many studies. Preliminary psychometric data on the scale revealed an internal consistency reliability of 0.89 and significant Pearson’s correlation coefficients between dimensions of the scale with positive correlation to one another [18-19].^ ^Respondents are asked to rate the level of agreement on a 7-point Likert scale (from 1 = strongly disagree to 7 = strongly agree) with statements such as *“Patients feel better when physicians understand their feelings."* Negatively worded items were reverse-scored. Final scores can range from 20 to 140. In the M1 sample, internal consistency (Cronbach’s alpha) equaled 0.73 for the pre-activity survey and 0.71 for the post-activity survey. A standardized Cronbach’s alpha also demonstrated good internal consistency (0.80 for the pre-activity survey and 0.77 for the post-activity survey). In the M4 sample, internal consistency (Cronbach’s alpha) equaled 0.67. A standardized Cronbach’s alpha demonstrated internal consistency equal to 0.63.

### Statistical analysis

A dependent samples t-test was used to examine medical student empathy scores pre- and post-activity for first-year medical students. Between-group differences (M1 vs. M4) were examined using independent samples t-test and nonparametric Mann-Whitney U test comparing full sample means (*n*_M1 _= 62, *n*_M4 _= 16) as well as equal sample size means using 16 randomly-selected M1 participants. A Pearson Correlation was used to evaluate the relationship between pre-activity and post-activity empathy scores for the M1 class. All statistical computations were completed with SPSS 22.0 (IBM; Chicago, IL) for Windows.

## Results

### Comparison of M1 pre-activity and post-activity JSPE:S scores

A total of 86 responses were received for the M1 class (73% response rate). Of these, 62 students completed both the pre-activity and post-activity surveys. These 62 responses were therefore used for statistical analysis.

**Figure 1:**
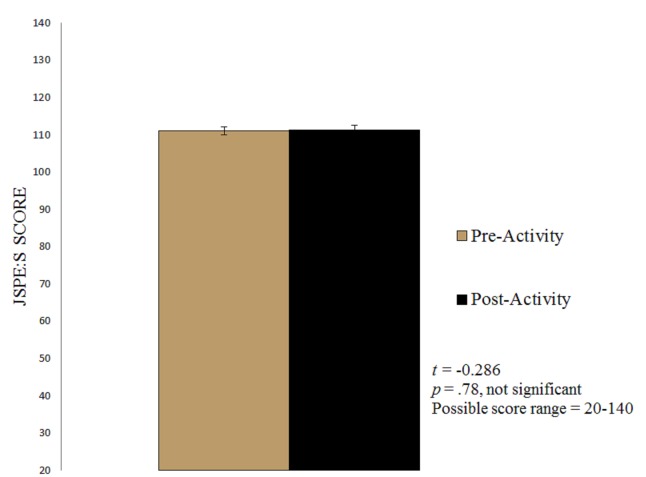
First-Year Medical Students' JSPE:S Scores Error bars represent Standard Error of the Mean (SEM). Pre-Activity SEM = 1.09; Post-Activity SEM = 1.16.

A dependent samples t-test revealed no statistically significant increase between pre-activity (*M *= 111.15, *SD *= 8.56) and post-activity (*M *= 111.38, *SD *= 9.12) empathy scores for the M1 class (*p *= .78) (Figure 1). A positive correlation was revealed to exist between pre- and post-activity empathy scores (*r *= 0.72, *p *< 0.001).

### Comparison of M1 and M4 JSPE:S scores

In order to compare the experimental group (M1) with the control group (M4), single-value mean imputation was used for survey question 9 (“*Attentiveness to patients’ experiences does not influence treatment outcome*”) to account for accidental omission of this question from the electronic survey sent to the M4 class only (see Discussion for rationale on handling missing data). Single-value mean imputation involves inputting the average for the observed data for that variable only and proceeding with standard analysis. For the item in question, the mean value for the observed answers (from the M1 pre-activity surveys) was 2.0, and thus, this value was used to reflect the data missing from question 9 on the M4 surveys. Descriptive statistics for M1 and M4 survey scores can be found in Table 1.

**Table 1:** Descriptive Statistics for JSPE:S Survey Results, M1 and M4 ^a^Pre Scores = Pre-Activity Scores; Post Scores = Post-Activity Scores

Measurement	M1 Pre Scores (M1 Post Scores)^a^	M4
Mean	111.15 (111.38)	106.56
Mode	116 (108)	None
Standard deviation	8.56 (9.12)	10.61
Standard error of the mean	1.09 (1.16)	2.65
Possible range/actual range	20-140/87-127 (20-140/88-136)	20-140/81-121
Cronbach’s alpha reliability	0.73 (0.71)	0.67

Empathy comparisons for the full sample M1 class (*n *= 62) and the M4 class (*n *= 16) revealed no statistically significant difference. Independent samples t-test between the post-activity experimental results and the M4 results yielded *p* = .11 (95% CI - 10.84-1.19) and nonparametric tests (Mann-Whitney U test) yielded *p* = .11.

Using Excel 2010 (Microsoft Corp., Redmond, WA), 16 random data sets were chosen from the 62 total participants from the M1 class and their post-activity survey scores were compared with survey scores of the 16 total participants from the M4 class in order to account for differences in sample size. This process was repeated to yield three randomly chosen groups of 16 students with no overlap between groups. Three separate data outputs (comparing each randomly chosen group of M1 scores with M4 scores) for independent samples t-test and non-parametric testing (Mann-Whitney U test) revealed no statistical significance (Table 2). 

**Table 2:** Equal Sample Size Comparison Outputs for Randomly-Selected M1 Scores vs. Full-Sample M4 Scores ^a^SD = standard deviation; SE = standard error; CI = confidence interval; M-W = Mann-Whitney

Sample	Mean	SD^a^	SE	*p *value	95% CI	M-W U *p *value
1	112.00	6.58	1.64	.09	-11.86 to 0.99	.14
2	113.13	7.82	1.96	.06	-13.32 to 0.19	.10
3	112.50	10.72	2.68	.13	-13.64 to 1.76	.10

## Discussion

### Previous studies of empathy

Many studies support the notion that empathy levels in medical students decline during their medical education [20-22]. These studies agree that this documented decline in empathy may jeopardize the quality of healthcare that patients receive and stress the need for longitudinal empathy studies, investigations into contributing factors, and improved measures for assessing empathy. Some authors have argued that the results of these studies are over-exaggerated and limited by sample size, response bias, and other challenges of studies of this type [23-25]

Several studies have suggested that educational interventions, such as the activity performed in this study, should play a role in increasing, maintaining, or slowing the documented decline in empathy over the four years in medical school [11-15]. Our results, at least in the short-term timeframe of this study, show no significant change in empathy levels within groups (M1; pre-activity vs. post-activity) or between groups (M1 vs. M4 classes), suggesting that alterations in a person's sense of empathy are developed and solidified over periods of months and years, rather than weeks. It is important to note that these results are based on a very small sample size, and therefore, it is difficult to draw conclusions about the impact of this activity on larger populations of medical students.

### Effectively measuring empathy

Still, other authors have argued that empathy is a difficult construct to measure, specifically, because there are two types of empathy: cognitive and emotional [26]. Although measures exist that independently measure each of these dimensions, the Jefferson Scale of Physician Empathy (JSPE) is the only measure available to evaluate empathy in medical students and physicians. However, the JSPE lacks psychometric data to measure both cognitive and emotional empathy independently, and some authors have already documented the limitations of the scale [18]. Even so, our research cannot comment on these proposed limitations as the restrictions of our own study (i.e., small sample size) limit the significance of our results as well as our ability to draw generalized conclusions. Nevertheless, in order to more accurately measure empathy, the suggestion arises for a validated scale with psychometric data to support the measurement of both the cognitive and emotional components of empathy as they relate to medical students and physicians.

### Violations of sample size

Regarding the violations of sample size and the large discrepancy in the response rate between control and experimental groups, we randomly selected 16 cases from the M1 class to compare with the 16 total responses from the M4 class. Analysis of three distinct randomized M1 samples showed no statistically significant difference in empathy scores (Table 2). Despite equalization of sample size, the small sample size remained a significant limitation to the study.

### Addressing missing data

A technical error in survey distribution to the M4 class resulted in missing data for one of the survey questions. The handling of missing data has been a widely discussed topic in the statistical literature, given its prevalence in the field of research. One of the most widely used techniques for addressing missing data is single-value mean imputation, also referred to as *mean substitution *[27].^ ^Mean substitution involves replacing missing data with the average for the observed data for that particular variable (i.e., inputting the mean for question 9 from the M1 pre-activity surveys for the missing data in the M4 surveys). Of course, any estimation technique has its limitations and can never fully compensate for data otherwise collected from experimental subjects. However, we felt that single-value mean imputation was the most effective technique to handle our missing data based on the ability to include all of the available cases in statistical analysis and because removal of the response in question from the M1 survey would significantly alter the results of the JSPE:S. Although mean substitution is a widely accepted method of handling missing data, its limitations deserve mention. Imputation of the mean results in bias in variance since observed values would likely stray from the mean [28-29].^ ^Because of this bias, we did not include variance data in our results.

### Small group discussion

Despite a lack of statistically significant numerical results from JSPE:S surveys, reviewing anonymous comments made by students on the JSPE:S and speaking with faculty members following the small group discussions revealed that many students were in fact impacted by the exercises (see Appendix E: Anonymous Student Comments). Many students expressed that following the regimen (specifically, making the lifestyle changes) would have been easier had they been provided with more information on how to count carbs, what they were allowed to eat, and why it was important for them to make these changes. Other students mentioned that because they did not “feel sick” they were less likely to remember to take their “medication,” translating their experience to a patient who is asymptomatic but whose disease can lead to severe complications if not treated. Both of these statements reflect the importance of patient education as it relates to compliance. Many students also expressed the impact that outside influences and busy schedules can have on patient compliance and felt an increased understanding for patients who are unable to strictly follow a physician’s orders.

Several students also provided suggestions for future studies, such as providing more of an incentive so that the study is taken more seriously, incorporating actual patients into the small-group discussions to describe their experiences, and asking students to follow the regimen for a longer duration of time.

### Future studies

It is important to note that the goal of this project was longitudinal in nature, but we were unable to perform a comparison across the years due to the low response rate. We would also like to stress the need for longitudinal studies of larger sample sizes in order to draw valid and well-supported conclusions that will aid in the development of curriculum adjuncts to help slow the decline of empathy if it indeed exists. It is possible that a longitudinal study that follows the M1 class may reveal a significant maintenance of empathy when compared to the M4 class who completed the curriculum without any educational activity. However, due to resource limitations and poor response rate, a longitudinal study was deferred at this time. We encourage future researchers with access to larger sample sizes to develop a protocol that will evaluate the longitudinal trend of empathy levels from the M1 year to the M4 year.

## Conclusions

Recognizing the correlation between physician empathy levels and patient care outcomes is critical to the development or modification of medical school curricula. Although other studies, such as the one performed by Clore and Jeffrey in which medical students assumed the role of a disabled patient [12], have suggested that role-playing activities similar to those employed in this study should have a significant effect on empathy levels, we believe that our sample size restriction and lack of longitudinal follow-up limited the significance of our results. Large-scale longitudinal studies should be conducted to evaluate the effects of simulated patient-role activities on maintaining or improving empathy levels in medical students and to explore alternative measures and activities to enhance the medical school curriculum and to train more compassionate, effective physicians.

## Appendices

### Appendix A: Pre-Activity Student Questionnaire

1. What is your gender?

            a. Male

            b. Female

**Instructions:** Please indicate your level of agreement or disagreement with each statement by selecting the appropriate circle. A higher number on the 7-point scale indicates more agreement.

2. Physician’s understanding of their patients’ feeling and the feelings of their patients’ family does not influence medical or surgical treatment.

            1                  2                  3                  4                       5                      6                           7

Strongly Disagree                                                                                                            Strongly Agree

3. Patients feel better when their physicians understand their feelings.

            1                  2                  3                  4                       5                      6                           7

Strongly Disagree                                                                                                            Strongly Agree

4. It is difficult for a physician to view things from patients’ perspectives.

            1                  2                  3                  4                       5                      6                           7

Strongly Disagree                                                                                                            Strongly Agree

5. Understanding body language is as important as verbal communication in the physician-patient relationship.

            1                  2                  3                  4                       5                      6                           7

Strongly Disagree                                                                                                            Strongly Agree

6. A physician’s sense of humor contributes to better clinical outcome.

            1                  2                  3                  4                       5                      6                           7

Strongly Disagree                                                                                                            Strongly Agree

7. Because people are different, it is difficult to see things from patients’ perspectives.

            1                  2                  3                  4                       5                      6                           7

Strongly Disagree                                                                                                            Strongly Agree

8. Attention to patients’ emotions is not important in history taking.

            1                  2                  3                  4                       5                      6                           7

Strongly Disagree                                                                                                            Strongly Agree

9. Attentiveness to patients’ experiences does not influence treatment outcome.

            1                  2                  3                  4                       5                      6                           7

Strongly Disagree                                                                                                            Strongly Agree

10. Physicians should try to stand in their patients’ shoes when providing care for them.

            1                  2                  3                  4                       5                      6                           7

Strongly Disagree                                                                                                            Strongly Agree

11. Patients value a physician’s understanding of their feelings in which is therapeutic in its own right.

            1                  2                  3                  4                       5                      6                           7

Strongly Disagree                                                                                                            Strongly Agree

12. Patients’ illnesses can be cured only by medical or surgical treatment; therefore, physicians’ emotional ties with their patients do not have a significant influence in medical or surgical treatment.

            1                  2                  3                  4                       5                      6                           7

Strongly Disagree                                                                                                            Strongly Agree

13. Asking patients about what is happening in their personal lives is not helpful in understanding their physical complaints.

            1                  2                  3                  4                       5                      6                           7

Strongly Disagree                                                                                                            Strongly Agree

14. Physicians should try to understand what is going on in their patients’ minds by paying attention to their non-verbal cues and body language.

            1                  2                  3                  4                       5                      6                           7

Strongly Disagree                                                                                                            Strongly Agree

15. I believe that emotion has no place in the treatment of medical illness.

            1                  2                  3                  4                       5                      6                           7

Strongly Disagree                                                                                                            Strongly Agree

16. Empathy is a therapeutic skill without which the physician’s success is limited.

            1                  2                  3                  4                       5                      6                           7

Strongly Disagree                                                                                                            Strongly Agree

17. Physicians’ understanding of the emotional status of their patients, as well as that of their families is one important component of the physician-patient relationship.

            1                  2                  3                  4                       5                      6                           7

Strongly Disagree                                                                                                            Strongly Agree

18. Physicians should try to think like their patients in order to better render care.

            1                  2                  3                  4                       5                      6                           7

Strongly Disagree                                                                                                            Strongly Agree

19. Physicians should not allow themselves to be influenced by strong personal bonds between their patients and their family members.

            1                  2                  3                  4                       5                      6                           7

Strongly Disagree                                                                                                            Strongly Agree

20. I do not enjoy reading non-medical literature or the arts.

            1                  2                  3                  4                       5                      6                           7

Strongly Disagree                                                                                                            Strongly Agree

21. I believe that empathy is an important therapeutic factor in medical treatment.

            1                  2                  3                  4                       5                      6                           7

Strongly Disagree                                                                                                            Strongly Agree

### Appendix B: Post-Activity Student Questionnaire

1. What is your gender?

            a. Male

            b. Female

2. To what extent did you complete or participate in the role-play activity?

            a. Not at all (0%)

            b. Less than half the time (<50%)

            c. About half the time (50%)

            d. More than half the time (>50%)

            e. Fully (100%)

3. To what extent did this activity contribute towards your understanding of the challenges faced by patients with medication and lifestyle modification adherence problems?

            a. Not at all

            b. To some extent

            c. Greatly

4. Was the role-play activity and discussion group a useful learning activity?

            a. Yes

            b. No

**Instructions:** Please indicate your level of agreement or disagreement with each statement by selecting the appropriate circle. A higher number on the 7-point scale indicates more agreement.

5. Physicians’ understanding of their patients’ feelings and the feelings of their patients’ family does not influence medical or surgical treatment.

            1                  2                  3                  4                       5                      6                           7

Strongly Disagree                                                                                                            Strongly Agree

6. Patients feel better when their physicians understand their feelings.

            1                  2                  3                  4                       5                      6                           7

Strongly Disagree                                                                                                            Strongly Agree

7. It is difficult for a physician to view things from patients’ perspectives.

            1                  2                  3                  4                       5                      6                           7

Strongly Disagree                                                                                                            Strongly Agree

8. Understanding body language is as important as verbal communication in the physician-patient relationship.

            1                  2                  3                  4                       5                      6                           7

Strongly Disagree                                                                                                            Strongly Agree

9. A physician’s sense of humor contributes to better clinical outcome.

            1                  2                  3                  4                       5                      6                           7

Strongly Disagree                                                                                                            Strongly Agree

10. Because people are different, it is difficult to see things from patients’ perspectives.

            1                  2                  3                  4                       5                      6                           7

Strongly Disagree                                                                                                            Strongly Agree

11. Attention to patients’ emotions is not important in history taking.

            1                  2                  3                  4                       5                      6                           7

Strongly Disagree                                                                                                            Strongly Agree

12. Attentiveness to patients’ experiences does not influence treatment outcome.

            1                  2                  3                  4                       5                      6                           7

Strongly Disagree                                                                                                            Strongly Agree

13. Physicians should try to stand in their patients’ shoes when providing care for them.

            1                  2                  3                  4                       5                      6                           7

Strongly Disagree                                                                                                            Strongly Agree

14. Patients value a physician’s understanding of their feelings in which is therapeutic in its own right.

            1                  2                  3                  4                       5                      6                           7

Strongly Disagree                                                                                                            Strongly Agree

15. Patients’ illnesses can be cured only by medical or surgical treatment; therefore, physicians’ emotional ties with their patients do not have a significant influence in medical or surgical treatment.

            1                  2                  3                  4                       5                      6                           7

Strongly Disagree                                                                                                            Strongly Agree

16. Asking patients about what is happening in their personal lives is not helpful in understanding their physical complaints.

            1                  2                  3                  4                       5                      6                           7

Strongly Disagree                                                                                                            Strongly Agree

17. Physicians should try to understand what is going on in their patients’ minds by paying attention to their non-verbal cues and body language.

            1                  2                  3                  4                       5                      6                           7

Strongly Disagree                                                                                                            Strongly Agree

18. I believe that emotion has no place in the treatment of medical illness.

            1                  2                  3                  4                       5                      6                           7

Strongly Disagree                                                                                                            Strongly Agree

19. Empathy is a therapeutic skill without which the physician’s success is limited.

            1                  2                  3                  4                       5                      6                           7

Strongly Disagree                                                                                                            Strongly Agree

20. Physicians’ understanding of the emotional status of their patients, as well as that of their families is one important component of the physician-patient relationship.

            1                  2                  3                  4                       5                      6                           7

Strongly Disagree                                                                                                            Strongly Agree

21. Physicians should try to think like their patients in order to better render care.

            1                  2                  3                  4                       5                      6                           7

Strongly Disagree                                                                                                            Strongly Agree

22. Physicians should not allow themselves to be influenced by strong personal bonds between their patients and their family members.

            1                  2                  3                  4                       5                      6                           7

Strongly Disagree                                                                                                            Strongly Agree

23. I do not enjoy reading non-medical literature or the arts.

            1                  2                  3                  4                       5                      6                           7

Strongly Disagree                                                                                                            Strongly Agree

24. I believe that empathy is an important therapeutic factor in medical treatment.

            1                  2                  3                  4                       5                      6                           7

Strongly Disagree                                                                                                            Strongly Agree

25. Please use the space below to comment on the activity.

### Appendix C: Diabetes Mellitus Patient Scenario

You are a 44-year-old male/female who had no known current medical problems but recently went to your physician’s office for a check-up after experiencing polyuria (increased urination).

Past medical and surgical history:

-Appendectomy when you were about 7 years old for appendicitis

-Fractured radius when you fell out of a tree when you were 12 years old, recovered without surgery

Medications

-None

Allergies

-Penicillin, anaphylaxis

Social History

You currently work as a physician on a general surgical service. You have been married for the last 12 years and have 3 children: an 8-year-old daughter and twins (both boys) who are 11 years old.

You drink wine about four times a week with dinner, smoked about a pack a day when you were in college but quit in medical school, and have never used any illegal drugs.

You drink 3 cups of coffee each day to keep up with pace of your lifestyle and your lack of sleep (approximately 5-6 hours each night).

You often skip breakfast. Lunch typically consists of whatever is in the vending machine. Your significant other usually cooks dinner for you and the children but you often indulge in dessert.

You do not spend time exercising and whatever free time you have is often spent at the movies or watching television. Your closest friends are from work and your spouse is supportive although your extended work hours place stress on your marriage.

Family History

*Mother*: 70 years old, living with diabetes mellitus and hypertension; stroke at the age of 69, currently without disability

*Father*: 71 years old, living with diabetes mellitus, no other medical problems

*Brother*: 44 years old, no medical problems

Diagnosis

-Diabetes mellitus, type 2

Lifestyle Changes Recommended by Physician

-Moderate exercise 20 minutes three times a week

-Limit carbohydrates to 65 grams per meal (3 meals per day)

Prescribed Medications (Follow instructions on each bottle)

-Metformin 500 mg by mouth twice a day (side effects: nausea, vomiting, gas, diarrhea) [16]  

-Glyburide 2.5 mg by mouth daily (side effects: nausea, heartburn, weight gain) [17]

### Appendix D: Faculty Orientation

Session Learning Objectives:

            -Demonstrate understanding of possible causes for medication non-adherence

            -Understand the potential consequences of medication non-adherence

            -Discuss ways that students can help minimize patient non-adherence

Students’ Pre-Session Tasks:

-Students will have completed a week-long role-playing activity which will have involved a combination of medication and lifestyle changes resembling those of a diabetes mellitus patient

Questions to Guide Discussion – Role-Playing Activity (Experimental Group)

-Why is medication non-adherence an important topic to discuss? What are the possible consequences of medication non-adherence?

-What surprised you about your experience?

-What was easiest and most difficult about following your regimen and why?

-What would have helped you have better adherence?

-How can you apply this experience to your future careers as physicians?

### Appendix E: Anonymous Student Comments

Note: Comments edited only for grammatical clarity.

“I did not feel that the study really mimicked what a patient goes through. It was hard to put myself in the role.”

”I thought that it was very helpful in terms of understanding how difficult it can be to make potentially drastic lifestyle modifications in a very short time period. I think the biggest thing I took away from the activity is that it is important to set aside time to address patient concerns and questions when prescribing new medications because throughout the course of the activity I came up with quite a few questions I would have wanted answered before beginning.”

“[This study] helped me realize the difficulties in compliance to medical treatment.”

“Keeping to the prescribed diet and exercise, and even taking the 'pills' regularly, was much harder than I anticipated when I first heard about this experiment. I even found myself embarrassed to be taking my fake pills in public. On the other hand, a confounding variable would be my inability to willingly suspend my disbelief for this activity. However, for an asymptomatic patient, or one with mild symptoms, it would be understandable that they do not truly feel bad, or understand how dangerous their situation is, so they may adhere to the prescription as poorly as me.”

“This exercise was a great way for me to understand how hard it can be to live with a disease. It taught me to be more empathic and understanding of my patients and their needs.”

“I would have liked to hear more from my classmates about their opinions and discussion rather than have the facilitator of the MOP session talk for almost all of the session.”

“I think the activity is good in allowing some insight on what it is like to change one's lifestyle to match a treatment regimen. Would do again!”

“I thought it was a meaningful activity, however, it seemed like many of us didn't take it seriously. If you could put more incentive, then it will be a better activity.”

“I believe this would have been more helpful if actual patients had been brought into the small group discussions to describe their experiences, and the impact that the attitude of their physician has on them.”

“I think the fact that we didn't feel sick but we still needed to take medications is mirrored in many patients and we should have a better understanding of how difficult it is to be compliant to medications we don't believe is making a significant impact. My personal difficulty is remembering to take my second dose and figuring out how much time should be in between the doses. Busy and unpredictable schedules also interfere with compliance.”

“Should really have been more than 4 days because there are so many variables that could have affected this (ie. Relay for life was this weekend, parents/friends visiting, etc) that would otherwise have not been an issue. There was not enough time for people to even start to create a habit, or if they were able to follow the regimen, really see if they were capable of sticking to it for a few more days.”

“A good learning experience that helps you to understand the difficulty a patient faces when asked to change lifestyle.”

“I think it would have been beneficial to have received medication and lifestyle counseling. It may have helped with compliance to know the importance of why we were asked to make the particular changes.”

“The diet was the hardest part! Next year, it would be better to give a heads up about the activity before students have gone shopping for this week's food. It would also be helpful to have a resource about how to count carbs, since I had no idea what I was doing.”

“The activity was really eye-opening - sometimes it's so easy for us as future clinicians to forget what it's like to really be a patient. The lifestyle changes I thought were particularly difficult because I knew there were many resources (websites, apps, etc) that could help with counting carbs, but felt overwhelmed with what was the right/best way while also considering how complex certain meals are, which really made me empathize with patients with restricted diets and how difficult/overwhelming it can be to make lifestyle changes so quickly.”

“I think the one thing I learned from this activity is the drastic effect that external circumstances have on patient compliance. I had every intention of adhering to the regimen given to us in this simulation, and did a decent job the first few days, but unexpected circumstances happened in my life and taking my "medications" was put on the backburner. I also learned that I was more compliant when I was around other students who were compliant and when I set personal reminders for myself to take the medicine. The addition of the medicine was much easier to adjust for than the diet and exercise expectations.”
